# Visualizing the phenotype diversity: a case study of Alexander disease

**DOI:** 10.5808/gi.21016

**Published:** 2021-09-30

**Authors:** Eisuke Dohi, Ali Haider Bangash

**Affiliations:** 1 Department of Neuroscience of Disease, Brain Research Institute, Niigata University, Niigata 951-8122, Japan; 2 Shifa College of Medicine, Shifa Tameer-e-Millat University, Islamabad 46000, Pakistan; 3 COST Action EVidence-Based RESearch (EVBRES), Western Norway University of Applied Sciences, Bergen 5063, Norway

**Keywords:** annotation, biomedical text mining, case reports, phenotype, rare diseases, symptoms

## Abstract

Since only a small number of patients have a rare disease, it is difficult to identify all of the features of these diseases. This is especially true for patients uncommonly presenting with rare diseases. It can also be difficult for the patient, their families, and even clinicians to know which one of a number of disease phenotypes the patient is exhibiting. To address this issue, during Biomedical Linked Annotation Hackathon 7 (BLAH7), we tried to extract Alexander disease patient data in Portable Document Format. We then visualized the phenotypic diversity of those Alexander disease patients with uncommon presentations. This led to us identifying several issues that we need to overcome in our future work.

## Introduction

Since only a relatively small number of patients have a rare disease, not all clinicians experience such patients, and not many experts experience a significant number of these patients. Such expert clinicians can experience so few of these patients that they cannot reach a comprehensive understanding of each rare disease. We clinicians, then, continue to further our knowledge through learning from the textbooks and previous publications. In doing so, we tend to learn only about the typical phenotype or the most well-known subtypes of a rare disease. In the real-life clinical setting, however, not every patient presents with typical symptoms, and their phenotypes are often diverse. Seeing patients with uncommon presentations of rare diseases is one of the reasons why we clinicians misdiagnose. This is a challenging issue, one in which we must make sure we do not overlook such patients, especially those whose delay of diagnosis could result in negative prognoses and even their lives being threatened.

## On-site Issues for Clinicians: Interpreting the Symptoms of Patients with Rare Diseases

Recent advances in diagnostic support tools allow us to develop better differential diagnosis lists based on a patient’s symptoms [1]. For example, PubCaseFinder includes information on different phenotypes that are associated with a disease. These have been extracted from the titles and abstracts of entire case reports found in PubMed [2]. In addition to these support tools, the cost of whole-genome sequencing is getting progressively cheaper. With this in mind, in the near future, we may encounter more patients than at present who show uncommon presentations with genetic mutations. Even now, in the case reports, we can find uncommon presentations in both common and uncommon diseases. Also, in the clinical practice, we clinicians sometimes find difficulty in interpreting the genetic results to determine whether these symptoms are due to genetic mutations or not. In rare diseases, in particular, clinicians often do not know enough about the wide diversity of the disease phenotypes.

## Attempt to Visualize the Phenotype Diversity in Alexander Disease

To address this issue, we tried to visualize the phenotype diversity in Alexander disease. The reasons why we chose Alexander disease are as follows: 1, The majority of cases of Alexander disease are caused by a genetic mutation of the *GFAP* gene [3]; 2, There are a manageable number of reported Alexander disease cases (less than 1,000); 3, There is a diversity in the age of onset, severity, and combination of symptoms; 4, There is an established feature-based disease classification for Alexander disease; and 5, Genotype-phenotype correlations have been examined [4]. Based on the established previous knowledge, we planned to validate, compare, and analyze the results of our method.

The outline of our plan to visualize the phenotype diversity in Alexander disease is shown in the accompanying figure. Medical Subject Headings 2021 (MeSH) Browser was used to search for the primary descriptors identifying Alexander disease in PubMed-indexed articles [5]. The MeSH term “Alexander Disease” was retrieved by an advanced search of PubMed [6]. We filtered the search results with “case reports” and obtained 139 case reports (as of 19 January 2021). Of the 139 case reports, 116 PDF files were available. We downloaded these and converted the entire text of all PDF files to text data with Apowersoft PDF Converter [7]. After ragged alignments were manually corrected, we annotated the extracted text data with Pubannotation [8]. For the annotation, the Human Phenotype Ontology (HPO) dictionaries on Pubdictionaries [9] were applied. Due to several problems, we could not proceed further in the process using automated means. So, we changed our direction toward listing the problems that we need to overcome.

## Discussion and Future Direction

**Fig. 1.**
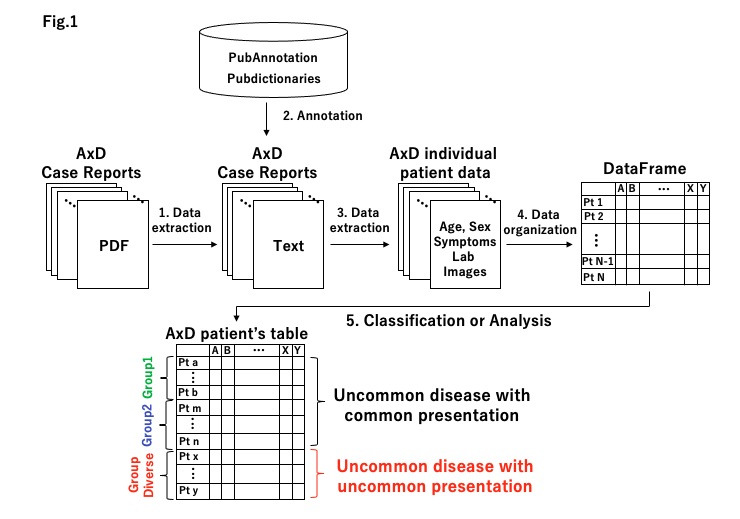
Workflow of individual patient data extraction from case report PDF for the visualization of uncommon disease with uncommon presentations. Step 1: Data extraction from PDF. Step 2: Annotation of data extraction with PubAnnotation and Pubdictionaries. Step 3: Data extraction from annotated text data as individual patient data. Step 4: Generation of the DataFrame with individual patient and clinical data (such as symptoms, lab images, and genetic mutation). Step 5: Classification of the patients based on their combination of phenotypes. AxD, Alexander disease.

Regarding the collecting of case reports of Alexander disease patients, we could not access the total number of reported cases (more than 500) [4]. One of the reasons for this was that we could not extract patient data from a particular type of publication, namely case series. Case series usually provide patient data in table format, and issues occurred when we extracted patient data from tables in PDF form. In step 1 of the figure, data extraction from PDF has several issues, but novel methods and algorithms [10] are being developed. We need to optimize our process in order to make automated data extraction possible. In step 2 (annotation), it was hoped that by using Pubdictionaries along with the HPO we would be able to annotate the technical terms related to the disease. However, a number of technical words, especially those describing neurological and psychiatric symptoms, were not annotated. It will be necessary to improve the dictionary with the help of expert clinicians from each area of expertise. Interestingly, we found that clinicians sometimes used non-technical terms to describe patients, even in scientific papers, and these were not annotated. For example, the term “occasional fall” is used to describe “unsteadiness,” and such words sometimes do not show up in the medical dictionaries. Also, the majority of symptoms should be interpreted in a context-dependent manner. For example, in “He became verbally abusive, introverted and aggressive, and had little insight into his condition,” the underlined part of the sentence describes the disease progression and severity. Symptoms, unlike other objective clinical parameters such as laboratory or radiographic tests, tend to be described in subjective and descriptive manners. We need, then, to apply natural language processing and develop more powerful dictionaries with the help of experienced clinicians. In step 3, we could not find an adequate tool for extracting “individual patient data.” Since paragraphs and locations of patient data are not structured between each paper and each journal, an algorithm and novel dictionaries would be required to extract and restructure “individual patient data.” We are currently developing such tools and hope to be soon able to extract and restructure this data. We had not reached this point in step 4. We noticed, however, that if we try to collect all of the patient features, then the number of features might be relatively large compared with the number of patients, especially for rare diseases. Although symptoms may be the same, the number of features would be multiplied if we try to include the severity of each symptom. The same thing would happen if we decided to include the time course of newly emerged symptoms and the progression of each symptom along with the disease progression. Through steps 1 to 4, we will successfully establish the “symptom-DataFrame,” which will be indexed with individual Alexander disease patients (rows) and their symptoms (columns) (Fig. 1). The columns could be expanded with other data such as lab data, image data, and any individual clinical information. In step 5, we will attempt to classify the individual patient with the disease. We can get the patient’s DataFrame using the established patient’s classification. Alternatively, we can apply a machine learning approaches to classify the relevant patient features. We will explore this further once we develop the “symptom-DataFrame.”

As mentioned above, there are problems that need to be overcome. However, the solving of several problems is already ongoing using multiple approaches, including the testing of a suitable PDF data extraction method [10], the development of new dictionaries, the application of validated natural language processing [11], testing the effect of limiting the relevant patient features, and application of machine learning to explore the relevant patient features. In addition to these, while the structured recording and storing of patient data for all diseases will need to be carried out consistently in the future, we need to seriously think about a more flexible way of doing this, as the sheer volume of data from individual patients is increasing massively in this era of big data. With this in mind, we would like to emphasize that seamless collaboration between informaticians and clinicians will become increasingly important to develop such "useful tools" and get clinicians to use them. We thoroughly enjoyed this attempt during Biomedical Linked Annotation Hackathon 7 (BLAH7), and we hope many more clinicians will be interested in this field and join with informaticians to develop and apply “useful tools.” Once we develop this automated kind of system, we will be able to analyze patient data from rare diseases that have not been well investigated. It will also, of course, be helpful to understand the individual patients not only for clinicians but for the patients themselves and their families.
